# Analysis and prediction of disease burden related to opioid use disorder in China based on GBD 2021

**DOI:** 10.3389/fpubh.2025.1680854

**Published:** 2025-11-24

**Authors:** RuiYan Wang, Yujia Liu, XingYu Wan, Tianhu Liang, Junxian Zhao, Hao Wang, Yan Ma, Yaolong Chen, Xinghua Lv

**Affiliations:** 1 The First School of Clinical Medicine, Lanzhou University, Lanzhou, China; 2 Research Centre for Clinical Medicine, The First Hospital of Lanzhou University, Lanzhou, China; 3 Research Unit of Evidence-Based Evaluation and Guidelines, Chinese Academy of Medical Sciences (2021RU017), School of Basic Medical Sciences, Lanzhou University, Lanzhou, China; 4 Day Surgery Centre, The First Hospital of Lanzhou University, Lanzhou, China

**Keywords:** disease burden, opioid drugs, drugs use disorder, anesthesia, public health, epidemiology

## Abstract

**Objective:**

To analyze the disease burden of opioid use disorder (OUD) in China from 1990 to 2021 and predict its future trends.

**Methods:**

Annual data from the Global Burden of Disease (GBD 2021) database were used to describe the disease burden through metrics such as prevalence, incidence, mortality, and disability-adjusted life years (DALYs). Joinpoint regression models were employed to analyze trends in disease burden. The estimated annual percentage change (EAPC) was calculated to quantify the temporal trends in age-standardized rates (ASRs). The age-period-cohort (APC) model explored age, period, and cohort effects on the OUD burden. Health inequality analyses examined the relationship between the OUD burden and the Sociodemographic Index (SDI). The autoregressive integrated moving average (ARIMA) model was used to forecast the OUD disease burden over the next 15 years.

**Results:**

Compared to 1990, the OUD-related disease burden in China declined across various indicators by 2021, with a significant decrease in the age-standardized mortality rate (EAPC = –5.26). Meanwhile, the global disease burden of OUD increased. In China, females, individuals aged 15–24 years, and recent birth cohorts showed higher disease burdens. Over time, the relationship between DALYs and SDI gradually weakened. Projections suggest that the burden of OUD in China is expected to increase over the next 15 years.

**Conclusion:**

Opioid misuse is a significant factor contributing to the global disease burden and challenges in anesthetic and surgical care. Effective measures are urgently needed to reduce the burden of opioid dependence and promote advancements in pain-free medical care.

## 1 Background

Substance use disorders (SUDs) refer to the compulsive and non-medical use of dependence-inducing substances (e.g., opioids, cocaine, cannabis) for their psychoactive effects. This behavior contributes to serious individual health problems, public health burdens, and societal consequences (1, 2). Research indicates that substance use disorders ranked among the top three contributors to global years lived with disability (YLD) in 2021. Moreover, between 2010 and 2021, the age-standardized YLD rate attributable to SUDs demonstrated the most significant increase among all leading causes (3). According to reports, over 2.5 billion people worldwide use drugs, with more than 60 million affected by opioid use disorders. Among all SUDs, opioid use disorder (OUD) is the most lethal, accounting for approximately two-thirds of drug-related deaths worldwide (4).

Opioids, derived from the poppy plant (*Papaver somniferum*) and its semi-synthetic or synthetic derivatives, are widely recognized for their analgesic, sedative, and euphoric properties. As one of the most commonly used pain relievers in clinical anesthesia, they also carry significant risks when misused, including severe health deterioration and fatal overdoses (5, 6). In recent years, the increasing number of malignant tumor patients in China has been accompanied by a rising trend in opioid misuse, particularly concerning the use and abuse of oral opioids (7). Managing perioperative anesthesia and pain control in patients with opioid use disorder has become a critical challenge in clinical practice, directly influencing the progress of pain-free healthcare (8). Opioid misuse is now a major focus for national regulatory authorities (9). Although previous studies (10, 11)have addressed the global burden of substance use disorders, a comprehensive investigation into the prevalence and burden of opioid use within China remains lacking.

This study aims to utilize the GBD (Global Burden of Disease) database to conduct a comprehensive statistical analysis of the latest disease burden data related to OUD from 1990 to 2021 (3). The analysis classifies the data by gender, age, and time period to provide a detailed overview of the disease burden associated with OUD over the past three decades. Additionally, it forecasts the burden from 2020 to 2035, with the goal of offering valuable insights for OUD prevention strategies and contributing to the advancement of pain-free healthcare in China.

## 2 Methods

### 2.1 Data sources

Data on OUD from 1990 to 2021 were obtained from the Institute for Health Metrics and Evaluation (IHME) official website using the GBD Results Tool.^1^ The dataset included incidence, prevalence, mortality, and disability-adjusted life years (DALYs), along with corresponding crude rates (CRs) and age-standardized rates (ASRs: ASIR; ASPR; ASMR; ASDR). These metrics were categorized by gender (male and female), age (15 groups spanning ages 15 to ≥95, in 5-year intervals), and geographic levels (China and global). All data estimates were provided with 95% uncertainty intervals (95% UI). To forecast future trends of OUD, population forecast data for 2020–2035 were also retrieved from a public database^2^ to project the ASR of OUD in China for the same period.

### 2.2 Statistics description

OUD burden was defined per ICD-10 codes F11.0-F11.9. Mortality and DALYs were age-standardized using the GBD reference population. DALYs were used to estimate the prevalence and disease burden of substance dependence, and the annual percentage change (EAPC) was calculated to describe ASR trends over specific periods, assessing the long-term trend in OUD burden. It was assumed that the natural logarithm of ASR has a linear relationship with time, expressed as y = *α* + *β*x + ɛ, where y = ln (rate), x is the calendar year, and ε represents the error term. The EAPC was calculated as EAPC = 100 × (exp(*β*) − 1), with its 95% UI derived from the model. ASR was considered to be increasing if the 95% UI of EAPC was above zero, decreasing if below zero, and stable if it included zero. A *p*-value <0.05 was deemed statistically significant (12). Estimates are presented as absolute numbers and age-standardized rates per 100,000 population, with a 95% UI, rounded to two decimal places.

### 2.3 Statistical analysis

#### 2.3.1 Joinpoint regression analysis

The Joinpoint regression model in R was used to analyze trends in ASRs for OUD. Initially, a linear regression model was fitted to capture the overall trend. Subsequently, joinpoints were sequentially added along the time axis, dividing the study period into intervals for trend fitting to identify points of trend changes. The Akaike Information Criterion (AIC) was used with each added joinpoint to evaluate model fit, determining the optimal number of joinpoints to balance complexity and goodness of fit. The final model, with the lowest AIC, was selected as the optimal model, and the joinpoint locations were extracted. After model fitting, the annual percentage change (APC) and its 95% UI were calculated for each interval to describe the significance of trends, with the average annual percent change (AAPC) used to summarize the overall trend. The AAPC with a 95% UI above zero indicated an increasing ASR trend, while a value below zero indicated a decreasing trend. A *p*-value < 0.05 was considered statistically significant for each identified trend change.

#### 2.3.2 Age-period-cohort model

The Age-Period-Cohort (APC) model is widely used in sociological and epidemiological studies. Based on a Poisson distribution, the APC model reflects time trends in incidence or mortality by age, period, and cohort. Age effects indicate differences in OUD prevalence across age groups due to age-related factors. Period effects represent changes in OUD prevalence due to human factors, such as advancements in diagnostics. Cohort effects reflect variations in OUD prevalence across birth cohorts due to differential exposure to risk factors. Data on incidence and mortality rates for each five-year age group from 1990 to 2021, along with annual population estimates, were obtained from the GBD database. Incidence or death counts and cumulative incidence and mortality rates across age groups were calculated. The APC model was fitted using the appropriate R (4.3.2) package, with the best model determined by comparing residuals.

#### 2.3.3 Measurement health inequalities

The Slope Index of Inequality (SII) and Concentration Index (CI) were used to assess inequality related to the Social Development Index (SDI) across countries (13). SII was calculated by regressing age-standardized DALYs rates related to OUD on a relative income-based social rank, determined by the midpoints of cumulative population intervals ranked by per capita GDP. CI was estimated by fitting a Lorenz curve to the cumulative distribution of the population ranked by income and their corresponding OUD-related DALYs burden, providing a summary measure of health inequality (14, 15).

#### 2.3.4 ARIMA forecast model

The autoregressive integrated moving average (ARIMA) model, a commonly used method in time series analysis, was employed to forecast future trends in OUD (3). ARIMA effectively captures trends and seasonality in time series data by combining autoregressive (AR), differencing (I), and moving average (MA) components. Model parameters are denoted as ARIMA (*p, d, q*), where *p* indicates the order of the autoregressive term, *d* denotes the degree of differencing, and *q* refers to the order of the moving average term.

## 3 Results

### 3.1 China and global burden and trends

Using GBD 2021 data, the trends in OUD cases and ASRs from 1990 to 2021 were evaluated (Tables 1–5). In 2021, the nationwide number of incident cases, prevalent cases, deaths, and DALYs associated with OUD were 235.26 (95% UI: 194.46, 282.46), 1472.27 (95% UI: 1222.9, 1734.65), 5.74 (95% UI: 4.52, 7.09), and 849.54 (95% UI: 649.86, 1041.69), respectively. The corresponding age-standardized incidence rate (ASIR), prevalence rate (ASPR), mortality rate (ASMR), and DALYs rate (ASDR) were 16.66 (95% UI: 13.82, 20.22) per 100,000, 94.35 (95% UI: 77.16, 122.47) per 100,000, 0.57 (95% UI: 4.52, 7.09) per 100,000, and 53.97 (95% UI: 40.54, 66.99) per 100,000 (Table 1). Compared to 1990, these indicators showed significant decreases, with EAPCs of −1.96 (−2.02, −1.92), −2.27 (−2.41, −2.2), −5.26 (−5.24, −5.06), and −3.37 (−3.5, −3.21) (Tables 2–5). Globally, the burden of OUD exhibited varying degrees of increase, with EAPCs of 0.16 (0.19, 0.11), 0.81 (0.91, 0.73), 1.05 (1.26, 1.06), and 0.91 (1.03, 0.89) (Tables 2–5).

**Table 1:** ASRs of opioid use disorders in the China and total in 1990 and 2021, and the temporal trends from 1990 to 2021.

Measure	China			Global		
	ASRs per 100,000 No. (95% UI) in 1990	ASRs per 100,000 No. (95% UI) in2021	EAPC in ASRs No. (95% UI)	ASRs per 100,000 No. (95% UI) in 1990	ASRs per 100,000 No. (95% UI) in 2021	EAPC in ASRs No. (95% UI)
Incidence	30.78 (26.01, 36.93)	16.66 (13.82, 20.22)	−1.96 (−2.02, −1.92)	23.37 (19.58, 28.48)	24.54 (20.74, 29.48)	0.16 (0.19, 0.11)
Prevalence	192.51 (164.43, 223.98)	94.35 (77.16, 112.47)	−2.27 (−2.41, −2.2)	154.59 (131.06, 181.26)	198.49 (173.42, 227.22)	0.81 (0.91, 0.73)
Mortality	1.71 (1.38, 2)	0.32 (0.26, 0.4)	−5.26 (−5.24, −5.06)	0.86 (0.76, 0.93)	1.19 (1.12, 1.29)	1.05 (1.26, 1.06)
DALYs	156.25 (122.44, 184.48)	53.97 (40.54, 66.99)	−3.37 (−3.5, −3.21)	103.69 (81.83, 122.75)	137.15 (112.29, 161.39)	0.91 (1.03, 0.89)

**Table 2:** Number of incident cases and incidence rate of opioid use disorders in the China and total in 1990 and 2021, and the temporal trends from 1990 to 2021.

Characteristics	China					Global				
	1990		2021		1990–2021	1990		2021		1990–2021
	Incident cases No. ×10^3^ (95% UI)	ASIR per 100,000 No. (95% UI)	Incident cases No. ×10^3^ (95% UI)	ASIR per 100,000 No. (95% UI)	EAPC in ASIR No. (95% UI)	Incident cases No. ×10^3^ (95% UI)	ASIR per 100,000 No. (95% UI)	Incident cases No. ×10^3^ (95% UI)	ASIR per 100,000 No. (95% UI)	EAPC in ASIR No. (95% UI)
Overall	412.59 (341.87, 506.61)	30.78 (26.01, 36.93)	235.26 (194.46, 282.46)	16.66 (13.82, 20.22)	−1.96 (−2.02, −1.92)	1301.55 (1077.63, 1598.05)	23.37 (19.58, 28.48)	1942.53 (1643.34, 2328.36)	24.54 (20.74, 29.48)	0.16 (0.19, 0.11)
Sex										
Male	195.94 (161.63, 238.35)	28.73 (24.12, 34.13)	113.08 (94.32, 134.13)	15.84 (13.14, 19.1)	−1.9 (−1.94, −1.86)	662 (550.79, 802.87)	23.52 (19.78, 28.47)	1005.21 (855.93, 1190.67)	25.2 (21.46, 29.85)	0.22 (0.26, 0.15)
Female	216.65 (178.02, 266.77)	32.9 (27.65, 40.09)	122.17 (100.95, 148.4)	17.53 (14.27, 21.43)	−2.01 (−2.11, −2.0)	639.55 (526.91, 793.02)	23.15 (19.27, 28.41)	937.31 (788.73, 1136.32)	23.82 (19.95, 29.04)	0.09 (0.11, 0.07)
Age at diagnosis (year) *										
15–19	71.06 (48.58, 97.97)	56.1 (38.36, 77.35)	20.8 (13.11, 30.49)	27.85 (17.55, 40.83)	−2.23 (−2.49, −2.04)	250.02 (172.42, 342.21)	48.13 (33.19, 65.88)	361.9 (265.11, 471.16)	58 (42.49, 75.51)	0.6 (0.8, 0.44)
20–24	124.04 (89.67, 165.36)	93.97 (67.93, 125.27)	35.71 (24.65, 49.2)	48.8 (33.69, 67.24)	−2.09 (−2.24, −1.99)	399.78 (294.69, 532.73)	81.24 (59.89, 108.26)	567.83 (436.35, 713.65)	95.09 (73.07, 119.51)	0.51 (0.64, 0.32)
25–29	66.97 (44.97, 93.21)	60.95 (40.93, 84.82)	30.17 (20.12, 41.83)	34.89 (23.27, 48.36)	−1.78 (−1.81, −1.8)	220.69 (152.76, 299.03)	49.86 (34.51, 67.56)	315.97 (234.65, 405.46)	53.7 (39.88, 68.92)	0.24 (0.47, 0.06)
30–34	36.65 (21.13, 57.42)	41.53 (23.95, 65.07)	30.61 (17.36, 47.65)	25.27 (14.33, 39.33)	−1.59 (−1.64, −1.61)	115.1 (63.9, 192.85)	29.86 (16.58, 50.04)	178.81 (102.84, 293.29)	29.58 (17.01, 48.52)	−0.03 (0.08, −0.1)
35–39	32.1 (20.17, 46.98)	35.14 (22.09, 51.44)	21.41 (13.91, 32.41)	20.2 (13.13, 30.58)	−1.77 (−1.66, −1.66)	86.3 (54.28, 130.42)	24.5 (15.41, 37.02)	126.45 (81.84, 189.54)	22.55 (14.59, 33.79)	−0.27 (−0.18, −0.29)
40–44	20.95 (11.9, 32.63)	31.23 (17.73, 48.63)	15.82 (9.15, 24.99)	17.28 (10, 27.3)	−1.89 (−1.83, −1.85)	59.25 (34.51, 92.94)	20.68 (12.05, 32.44)	90.59 (53.12, 143.35)	18.11 (10.62, 28.66)	−0.43 (−0.41, −0.4)
45–49	15.36 (8.96, 22.89)	29.75 (17.35, 44.34)	18.22 (10.45, 26.63)	16.52 (9.47, 24.14)	−1.88 (−1.93, −1.94)	45.98 (27.06, 67.89)	19.8 (11.65, 29.24)	81.25 (47.95, 119.75)	17.16 (10.13, 25.29)	−0.46 (−0.45, −0.47)
50–54	12.52 (6.68, 19.32)	26.25 (14, 40.49)	8.2 (9.81, 27.44)	15.06 (8.12, 22.7)	−1.78 (−1.74, −1.85)	40.68 (22.38, 60.77)	19.14 (10.53, 28.59)	67.73 (36.52, 101.22)	15.22 (8.21, 22.75)	−0.74 (−0.8, −0.73)
55–59	9.13 (4.79, 15.43)	21.05 (11.05, 35.57)	14.21 (7.57, 22.71)	12.92 (6.89, 20.65)	−1.56 (−1.51, −1.74)	26.69 (15.04, 42.91)	14.41 (8.12, 23.17)	48.71 (27.64, 76.8)	12.31 (6.99, 19.41)	−0.51 (−0.48, −0.57)
60–64	7.34 (3.75, 11.86)	20.77 (10.61, 33.56)	8.56 (4.35, 13.75)	11.72 (5.96, 18.83)	−1.83 (−1.84, −1.85)	19.66 (9.32, 32.89)	12.24 (5.8, 20.48)	32.47 (15.59, 53.9)	10.15 (4.87, 16.84)	−0.6 (−0.56, −0.63)
65–69	6.91 (4.22, 10.21)	25.34 (15.46, 37.41)	8.83 (5.37, 12.95)	11.51 (6.99, 16.89)	−2.51 (−2.53, −2.53)	14.72 (8.84, 22.3)	11.91 (7.15, 18.04)	25.04 (15.21, 37.76)	9.08 (5.51, 13.69)	−0.87 (−0.84, −0.89)
70–74	5.06 (3.06, 7.46)	26.9 (16.26, 39.64)	5.95 (3.4, 8.92)	11.16 (6.39, 16.73)	−2.8 (−2.97, −2.74)	9.99 (5.74, 15.45)	11.8 (6.78, 18.24)	17.51 (9.57, 26.84)	8.5 (4.65, 13.04)	−1.05 (−1.21, −1.08)
75–79	2.86 (1.63, 4.27)	25.09 (14.32, 37.48)	3.53 (2.15, 5.14)	10.66 (6.48, 15.52)	−2.72 (−2.53, −2.8)	6.73 (3.82, 10.18)	10.94 (6.2, 16.55)	11.51 (6.79, 16.95)	8.73 (5.15, 12.85)	−0.73 (−0.6, −0.81)
80–84	1.22 (0.66, 1.88)	23.12 (12.44, 35.52)	2.03 (1.14, 3)	10.25 (5.77, 15.15)	−2.59 (−2.45, −2.71)	3.72 (2.07, 5.56)	10.51 (5.84, 15.71)	8.44 (5.08, 12.12)	9.64 (5.8, 13.84)	−0.28 (−0.02, −0.41)
85–89	0.35 (0.22, 0.52)	20.7 (13.24, 30.73)	0.9 (0.59, 1.3)	9.49 (6.21, 13.67)	−2.48 (−2.41, −2.58)	1.6 (1.05, 2.32)	10.56 (6.92, 15.38)	5.16 (3.63, 7.06)	11.29 (7.93, 15.43)	0.22 (0.44, 0.01)
90–94	0.06 (0.03, 0.09)	18.47 (10.68, 28.5)	0.25 (0.16, 0.37)	8.67 (5.31, 12.72)	−2.41 (−2.23, −2.57)	0.49 (0.32, 0.7)	11.48 (7.42, 16.44)	2.37 (1.73, 3.16)	13.27 (9.66, 17.67)	0.47 (0.85, 0.23)
95+	0.01 (0, 0.01)	18.02 (8.8, 32.46)	0.06 (0.03, 0.09)	8.67 (4.36, 14.74)	−2.33 (−2.24, −2.51)	0.14 (0.07, 0.23)	13.47 (7.01, 23.06)	0.77 (0.43, 1.28)	14.2 (7.95, 23.51)	0.17 (0.41, 0.06)

ASIR, age-standardized incidence rate; EAPC, estimated annual percentage change; No., number; UI, uncertainty interval. *: Crude incidence rate in each age group.

**Table 3:** Number of prevalent cases and prevalence rate of opioid use disorders in the China and total in 1990 and 2021, and the temporal trends from 1990 to 2021.

Characteristics	China					Global				
	1990		2021		1990–2021	1990		2021		1990–2021
	Prevalent cases No. ×10^3^ (95% UI)	ASPR per 100,000 No. (95% UI)	Prevalent cases No. ×10^3^ (95% UI)	ASPR per 100,000 No. (95% UI)	EAPC in ASPR No. (95% UI)	Prevalent cases No. ×10^3^ (95% UI)	ASPR per 100,000 No. (95% UI)	Prevalent cases No. ×10^3^ (95% UI)	ASPR per 100,000 No. (95% UI)	EAPC in ASPR No. (95% UI)
Overall	2397.76 (2019.08, 2833.94)	192.51 (164.43, 223.98)	1472.27 (1222.9, 1734.65)	94.35 (77.16, 112.47)	−2.27 (−2.41, −2.2)	8120.81 (6801.33, 9596.42)	154.59 (131.06, 181.26)	16164.9 (14133.1, 18431.5)	198.49 (173.42, 227.22)	0.81 (0.91, 0.73)
Sex										
Male	1068.45 (897.71, 1254.08)	166.96 (143.03, 193.44)	681.55 (579.2, 793.3)	86.63 (72.3, 102.07)	−2.09 (−2.18, −2.04)	4112.04 (3496.12, 4, 815)	154.58 (132.54, 180.63)	8176.22 (7238.34, 9254.56)	200.23 (176.76, 226.9)	0.84 (0.93, 0.74)
Female	1329.3 (1106.81, 1568.99)	218.94 (186.15, 254.5)	790.72 (646.48, 942.57)	102.29 (82.22, 124)	−2.42 (−2.6, −2.29)	4008.77 (3293.73, 4771.65)	153.99 (128.53, 181.17)	7988.66 (6896.38, 9215.84)	196.11 (168.4, 226.88)	0.78 (0.88, 0.73)
Age at diagnosis (year) *										
15–19	101.96 (69.62, 142.37)	80.5 (54.96, 112.4)	29.65 (18.87, 43.46)	39.71 (25.27, 58.2)	−2.25 (−2.48, −2.1)	362.23 (250.99, 497.59)	69.74 (48.32, 95.8)	539.79 (402.22, 704.38)	86.51 (64.46, 112.88)	0.7 (0.93, 0.53)
20–24	473.17 (334.45, 646.43)	358.46 (253.37, 489.72)	131.26 (88.37, 189.78)	179.38 (120.76, 259.36)	−2.21 (−2.36, −2.03)	1575.25 (1151.67, 2123.6)	320.12 (234.04, 431.55)	2427.33 (1895.54, 3118.14)	406.48 (317.43, 522.16)	0.77 (0.99, 0.62)
25–29	488.34 (372.74, 622.37)	444.39 (339.2, 566.36)	195.86 (143.42, 257.96)	226.48 (165.83, 298.28)	−2.15 (−2.28, −2.05)	1790.05 (1387.91, 2262.29)	404.42 (313.57, 511.11)	3081.39 (2537.62, 3759.83)	523.74 (431.32, 639.05)	0.84 (1.03, 0.72)
30–34	340.42 (257.57, 439.82)	385.77 (291.89, 498.42)	242.52 (176.7, 324.74)	200.17 (145.85, 268.04)	−2.09 (−2.21, −1.98)	1296.28 (1012.83, 1661.73)	336.33 (262.79, 431.15)	2697.92 (2246.89, 3291.17)	446.32 (371.71, 544.46)	0.92 (1.12, 0.76)
35–39	297.43 (231.2, 387.82)	325.63 (253.13, 424.6)	175.81 (132.55, 231.03)	165.91 (125.09, 218.03)	−2.15 (−2.25, −2.13)	939.81 (747.33, 1217.53)	266.81 (212.16, 345.65)	2031.39 (1682.42, 2487.43)	362.19 (299.97, 443.5)	0.99 (1.12, 0.81)
40–44	185.91 (136.5, 243.03)	277.08 (203.44, 362.23)	124.44 (87.73, 168.43)	135.94 (95.85, 184.01)	−2.27 (−2.4, −2.16)	610.77 (462.55, 774.91)	213.2 (161.46, 270.49)	1484.66 (1223.16, 1765.53)	296.78 (244.51, 352.93)	1.07 (1.35, 0.86)
45–49	130.75 (99.01, 170.42)	253.29 (191.8, 330.14)	131.59 (96.02, 174.91)	119.28 (87.03, 158.55)	−2.4 (−2.52, −2.34)	419.51 (322.2, 538.22)	180.67 (138.76, 231.79)	1167.53 (970.65, 1403.75)	246.57 (204.99, 296.46)	1.01 (1.27, 0.8)
50–54	111.99 (82.84, 149.31)	234.73 (173.64, 312.94)	132.11 (90.26, 180.63)	109.31 (74.68, 149.45)	−2.44 (−2.69, −2.36)	368.71 (274.86, 487.24)	173.45 (129.3, 229.21)	981.72 (778.05, 1208.82)	220.65 (174.87, 271.69)	0.78 (0.98, 0.55)
55–59	88.4 (66.14, 115.52)	203.83 (152.51, 266.36)	107.01 (76.47, 143.95)	97.33 (69.56, 130.93)	−2.36 (−2.5, −2.26)	268.54 (199.53, 345.81)	145 (107.74, 186.72)	710.36 (563.88, 874.89)	179.51 (142.49, 221.08)	0.69 (0.91, 0.55)
60–64	62.04 (45.21, 85.35)	175.58 (127.93, 241.53)	62 (42.62, 89.46)	84.93 (58.38, 122.54)	−2.32 (−2.5, −2.17)	192.79 (142.25, 263.83)	120.04 (88.57, 164.27)	428.42 (334.26, 551.16)	133.86 (104.44, 172.21)	0.35 (0.53, 0.15)
65–69	47.77 (36.71, 60.87)	175.09 (134.56, 223.12)	59.24 (42.28, 78.59)	77.24 (55.12, 102.46)	−2.61 (−2.84, −2.48)	123.82 (94.21, 159.67)	100.17 (76.21, 129.18)	262.22 (207.11, 335.62)	95.06 (75.08, 121.67)	−0.17 (−0.05, −0.19)
70–74	35.7 (25.86, 47.94)	189.74 (137.42, 254.74)	38.6 (27.83, 52.62)	72.43 (52.21, 98.74)	−3.06 (−3.07, −3.01)	78.92 (57.73, 106.29)	93.22 (68.18, 125.55)	153.59 (116.86, 201.86)	74.61 (56.77, 98.07)	−0.72 (−0.59, −0.79)
75–79	21.57 (16.28, 28.37)	189.54 (143.05, 249.31)	22.46 (16.78, 29.35)	67.83 (50.66, 88.62)	−3.26 (−3.29, −3.28)	51.81 (39.27, 68.13)	84.17 (63.8, 110.67)	85.58 (66.49, 108.34)	64.89 (50.41, 82.15)	−0.84 (−0.76, −0.96)
80–84	9.27 (6.68, 12.31)	174.97 (126.03, 232.32)	12.48 (8.98, 16.56)	63.05 (45.39, 83.66)	−3.24 (−3.24, −3.24)	27.07 (19.3, 36.12)	76.53 (54.56, 102.11)	57.14 (43.16, 72.88)	65.24 (49.28, 83.21)	−0.51 (−0.33, −0.66)
85–89	2.59 (1.97, 3.37)	153.8 (116.61, 199.87)	5.45 (4.16, 6.97)	57.21 (43.71, 73.14)	−3.14 (−3.12, −3.19)	11.13 (8.66, 14)	73.64 (57.31, 92.68)	33.77 (27.75, 40.81)	73.87 (60.69, 89.26)	0.01 (0.19, −0.12)
90–94	0.4 (0.3, 0.52)	129.26 (97.09, 169.09)	1.49 (1.13, 1.93)	50.65 (38.59, 65.76)	−2.98 (−2.93, −3.0)	3.26 (2.54, 4.14)	75.98 (59.27, 96.55)	16.39 (13.72, 19.44)	91.61 (76.71, 108.66)	0.61 (0.84, 0.38)
95+	0.05 (0.03, 0.06)	113.52 (78.53, 154.36)	0.3 (0.21, 0.4)	46.56 (33.01, 61.98)	−2.83 (−2.76, −2.9)	0.86 (0.63, 1.1)	84.25 (62.34, 108.5)	5.67 (4.56, 6.9)	104.1 (83.58, 126.52)	0.68 (0.95, 0.5)

ASPR, age-standardized prevalence rate; EAPC, estimated annual percentage change; No., number; UI, uncertainty interval. *: Crude incidence rate in each age group.

**Table 4:** Number of death cases and mortality rate of opioid use disorders in the China and total in 1990 and 2021, and the temporal trends from 1990 to 2021.

Characteristics	China									
	1990		2021		1990–2021	1990		2021		1990–2021
	Death cases No. ×10^3^ (95% UI)	ASMR per 100,000 No. (95% UI)	Death cases No. ×10^3^ (95% UI)	ASMR per 100,000 No. (95% UI)	EAPC in ASMR No. (95% UI)	Death cases No. ×10^3^ (95% UI)	ASMR per 100,000 No. (95% UI)	Death cases No. ×10^3^ (95% UI)	ASMR per 100,000 No. (95% UI)	EAPC in ASMR No. (95% UI)
Overall	18.99 (15.08, 22.34)	1.71 (1.38, 2)	5.74 (4.52, 7.09)	0.32 (0.26, 0.4)	−5.26 (−5.24, −5.06)	41.57 (36.92, 45.06)	0.86 (0.76, 0.93)	99.56 (92.95, 108.05)	1.19 (1.12, 1.29)	1.05 (1.26, 1.06)
Sex										
Male	14.06 (11.15, 17.06)	2.61 (2.1, 3.11)	4.59 (3.45, 5.97)	0.52 (0.39, 0.67)	−5.07 (−5.29, −4.83)	31.38 (28.03, 34.42)	1.31 (1.17, 1.43)	70.55 (66.34, 76.05)	1.71 (1.61, 1.84)	0.86 (1.04, 0.82)
Female	4.94 (3.51, 6.36)	0.88 (0.63, 1.13)	1.15 (0.88, 1.5)	0.13 (0.1, 0.18)	−5.98 (−5.76, −5.75)	10.19 (8.59, 11.94)	0.42 (0.36, 0.49)	29 (26.03, 32.26)	0.68 (0.61, 0.76)	1.57 (1.72, 1.43)
Age at diagnosis (year) *										
15–19	0.95 (0.65, 1.26)	0.75 (0.51, 0.99)	0.06 (0.04, 0.07)	0.08 (0.06, 0.1)	−6.97 (−6.67, −7.13)	1.88 (1.55, 2.24)	0.36 (0.3, 0.43)	1.73 (1.55, 1.94)	0.28 (0.25, 0.31)	−0.81 (−0.59, −1.05)
20–24	1.72 (1.21, 2.16)	1.3 (0.92, 1.63)	0.14 (0.11, 0.18)	0.2 (0.15, 0.25)	−5.86 (−5.68, −5.87)	4.09 (3.51, 4.56)	0.83 (0.71, 0.93)	5.97 (5.53, 6.49)	1 (0.93, 1.09)	0.6 (0.87, 0.51)
25–29	1.75 (1.25, 2.1)	1.59 (1.13, 1.91)	0.26 (0.2, 0.34)	0.3 (0.24, 0.39)	−5.24 (−4.88, −5.0)	5.09 (4.47, 5.5)	1.15 (1.01, 1.24)	9.89 (9.32, 10.49)	1.68 (1.58, 1.78)	1.23 (1.45, 1.17)
30–34	2.6 (2.04, 3.11)	2.94 (2.31, 3.52)	0.85 (0.68, 1.05)	0.7 (0.56, 0.87)	−4.52 (−4.47, −4.41)	6.11 (5.48, 6.65)	1.58 (1.42, 1.73)	13.24 (12.53, 14)	2.19 (2.07, 2.32)	1.06 (1.22, 0.95)
35–39	2.93 (2.28, 3.58)	3.21 (2.49, 3.92)	0.77 (0.6, 0.96)	0.72 (0.56, 0.91)	−4.71 (−4.7, −4.6)	5.87 (5.15, 6.51)	1.67 (1.46, 1.85)	13.3 (12.49, 14.15)	2.37 (2.23, 2.52)	1.14 (1.38, 1.0)
40–44	2.15 (1.67, 2.56)	3.2 (2.49, 3.81)	0.6 (0.46, 0.78)	0.66 (0.5, 0.85)	−4.96 (−5.05, −4.72)	4.3 (3.77, 4.74)	1.5 (1.32, 1.65)	11.86 (10.99, 12.85)	2.37 (2.2, 2.57)	1.49 (1.66, 1.44)
45–49	1.37 (1.06, 1.72)	2.65 (2.05, 3.33)	0.54 (0.39, 0.72)	0.49 (0.35, 0.66)	−5.3 (−5.54, −5.09)	2.81 (2.47, 3.15)	1.21 (1.06, 1.36)	10.04 (9.18, 11.11)	2.12 (1.94, 2.35)	1.83 (1.97, 1.78)
50–54	1.35 (1.05, 1.66)	2.84 (2.21, 3.48)	0.53 (0.4, 0.7)	0.44 (0.33, 0.58)	−5.84 (−5.95, −5.62)	2.7 (2.36, 3.04)	1.27 (1.11, 1.43)	8.97 (8, 10.15)	2.02 (1.8, 2.28)	1.51 (1.57, 1.52)
55–59	1.12 (0.89, 1.39)	2.59 (2.05, 3.2)	0.42 (0.32, 0.54)	0.38 (0.29, 0.49)	−6.0 (−6.11, −5.87)	2.3 (2.03, 2.56)	1.24 (1.1, 1.38)	8.22 (7.28, 9.36)	2.08 (1.84, 2.37)	1.68 (1.67, 1.76)
60–64	0.94 (0.76, 1.13)	2.66 (2.14, 3.21)	0.29 (0.23, 0.37)	0.4 (0.31, 0.51)	−5.93 (−6.04, −5.76)	1.89 (1.68, 2.1)	1.18 (1.05, 1.31)	5.31 (4.73, 6.04)	1.66 (1.48, 1.89)	1.11 (1.11, 1.19)
65–69	0.83 (0.68, 1)	3.06 (2.48, 3.65)	0.36 (0.28, 0.45)	0.46 (0.36, 0.58)	−5.93 (−6.04, −5.76)	1.59 (1.4, 1.77)	1.29 (1.13, 1.43)	3.59 (3.23, 4)	1.3 (1.17, 1.45)	0.02 (0.11, 0.04)
70–74	0.57 (0.45, 0.71)	3.03 (2.4, 3.77)	0.38 (0.28, 0.51)	0.72 (0.53, 0.96)	−4.53 (−4.76, −4.32)	1.06 (0.93, 1.22)	1.25 (1.1, 1.44)	2.37 (2.12, 2.62)	1.15 (1.03, 1.27)	−0.27 (−0.21, −0.4)
75–79	0.43 (0.35, 0.52)	3.78 (3.09, 4.56)	0.33 (0.25, 0.42)	1 (0.75, 1.27)	−4.2 (−4.46, −4.04)	0.88 (0.79, 0.98)	1.43 (1.28, 1.58)	1.65 (1.48, 1.83)	1.25 (1.13, 1.39)	−0.43 (−0.4, −0.41)
80–84	0.18 (0.14, 0.21)	3.39 (2.67, 4.05)	0.11 (0.08, 0.14)	0.53 (0.39, 0.71)	−5.81 (−6.02, −5.46)	0.56 (0.49, 0.62)	1.57 (1.4, 1.75)	1.33 (1.15, 1.49)	1.52 (1.32, 1.7)	−0.1 (−0.19, −0.09)
85–89	0.08 (0.06, 0.09)	4.68 (3.72, 5.56)	0.08 (0.06, 0.1)	0.83 (0.63, 1.05)	−5.43 (−5.57, −5.23)	0.29 (0.25, 0.32)	1.89 (1.64, 2.09)	1.06 (0.87, 1.19)	2.32 (1.9, 2.6)	0.66 (0.48, 0.71)
90–94	0.02 (0.01, 0.02)	6.02 (4.72, 7.17)	0.03 (0.02, 0.03)	0.88 (0.69, 1.13)	−6.01 (−6.01, −5.79)	0.11 (0.09, 0.12)	2.57 (2.15, 2.87)	0.72 (0.55, 0.82)	4 (3.1, 4.56)	1.44 (1.19, 1.5)
95+	0 (0, 0)	2.37 (1.83, 2.93)	0 (0, 0)	0.49 (0.37, 0.62)	−4.96 (−5.03, −4.89)	0.03 (0.03, 0.04)	3.18 (2.46, 3.64)	0.29 (0.21, 0.34)	5.23 (3.89, 6.19)	1.62 (1.49, 1.73)

ASMR, age-standardized mortality rate; EAPC, estimated annual percentage change; No., number; UI, uncertainty interval. *: Crude incidence rate in each age group.

**Table 5:** Number of DALYs cases and DALYs rate of opioid use disorders in the China and total in 1990 and 2021, and the temporal trends from 1990 to 2021.

Characteristics	China					Global				
	1990		2021		1990–2021	1990		2021		1990–2021
	DALYs cases No. ×10^3^ (95% UI)	ASDR per 100,000 No. (95% UI)	DALYs cases No. ×10^3^ (95% UI)	ASDR per 100,000 No. (95% UI)	EAPC in ASDR No. (95% UI)	DALYs cases No. × 103 (95%UI)	ASDR per100000 No. (95%UI)	DALYs cases No. × 103 (95%UI)	ASDR per100000 No. (95%UI)	EAPC in ASDR No. (95%UI)
Overall	1926.11 (1501.31, 2283.28)	156.25 (122.44, 184.48)	849.54 (649.86, 1041.69)	53.97 (40.54, 66.99)	−3.37 (−3.5, −3.21)	5415.25 (4, 242, 6437.81)	103.69 (81.83, 122.75)	11218.5 (9188.66, 13159.5)	137.15 (112.29, 161.39)	0.91 (1.03, 0.89)
Sex										
Male	1127.82 (896.53, 1320.95)	179.95 (145.17, 210.06)	474.14 (372.14, 575.24)	58.87 (45.81, 71.68)	−3.54 (−3.65, −3.41)	3290.02 (2691.25, 3829.09)	125.14 (102.6, 144.69)	6745.37 (5708.93, 7736.92)	164.33 (138.97, 188.54)	0.88 (0.98, 0.86)
Female	798.29 (589.01, 993.28)	131.79 (98.26, 161.44)	375.4 (270.18, 472.95)	48.89 (35.03, 62.21)	−3.15 (−3.27, −3.03)	2125.23 (1540.78, 2648.11)	81.86 (60.1, 101.53)	4473.15 (3467.34, 5448.69)	109.42 (84.49, 133.54)	0.94 (1.1, 0.89)
Age at diagnosis (year) *										
15–19	113.02 (83.23, 145.07)	89.23 (65.71, 114.53)	17 (10.71, 24.87)	22.76 (14.34, 33.31)	−4.31 (−4.79, −3.91)	292.02 (220.09, 370.96)	56.22 (42.37, 71.42)	355.92 (265.04, 451.43)	57.04 (42.48, 72.35)	0.05 (0.01, 0.04)
20–24	319.91 (230.93, 412.42)	242.36 (174.94, 312.44)	66.44 (42.01, 96.92)	90.8 (57.42, 132.44)	−3.12 (−3.53, −2.73)	947.17 (686.42, 1243.95)	192.48 (139.49, 252.79)	1426.24 (1060.23, 1830.82)	238.84 (177.55, 306.59)	0.7 (0.78, 0.62)
25–29	318.41 (233.6, 395.8)	289.76 (212.57, 360.18)	100.42 (68.71, 136.81)	116.12 (79.45, 158.19)	−2.91 (−3.12, −2.62)	1073.41 (793.29, 1352.95)	242.51 (179.22, 305.67)	1907.55 (1481.96, 2359.32)	324.22 (251.89, 401.01)	0.94 (1.1, 0.88)
30–34	293.32 (231.2, 357.42)	332.4 (262, 405.04)	151.97 (111.85, 198.62)	125.43 (92.32, 163.94)	−3.09 (−3.31, −2.88)	893.64 (706.18, 1107.51)	231.86 (183.22, 287.35)	1884.36 (1506.04, 2281.12)	311.73 (249.15, 377.37)	0.96 (1.0, 0.88)
35–39	279.86 (223.29, 342.08)	306.39 (244.46, 374.52)	114.58 (84.87, 151.21)	108.13 (80.09, 142.7)	−3.3 (−3.54, −3.06)	699.53 (556.76, 863.18)	198.59 (158.06, 245.05)	1537.46 (1259.36, 1861.14)	274.12 (224.54, 331.83)	1.05 (1.14, 0.98)
40–44	180.18 (142.13, 218.32)	268.55 (211.84, 325.4)	80.78 (59.47, 104.42)	88.25 (64.97, 114.08)	−3.53 (−3.74, −3.32)	457.19 (367.66, 558.62)	159.59 (128.34, 194.99)	1172.74 (976.52, 1379.53)	234.43 (195.21, 275.77)	1.25 (1.36, 1.12)
45–49	112.81 (87.83, 139.2)	218.54 (170.15, 269.68)	77.44 (55.25, 102.16)	70.2 (50.08, 92.6)	−3.6 (−3.87, −3.39)	291.56 (233.05, 364.99)	125.57 (100.37, 157.19)	902.29 (749.73, 1045.26)	190.56 (158.34, 220.75)	1.35 (1.48, 1.1)
50–54	97.42 (77.04, 119.57)	204.19 (161.47, 250.61)	74.33 (51.6, 102.2)	61.5 (42.7, 84.56)	−3.8 (−4.2, −3.44)	251.28 (195.92, 315.44)	118.21 (92.17, 148.39)	734.32 (595.89, 869.41)	165.04 (133.93, 195.41)	1.08 (1.21, 0.89)
55–59	73.31 (58.37, 88.92)	169.04 (134.59, 205.03)	57.2 (40.29, 76.46)	52.02 (36.65, 69.55)	−3.73 (−4.11, −3.43)	183.59 (141.03, 226.63)	99.13 (76.15, 122.37)	554.71 (455.05, 655.93)	140.17 (114.99, 165.75)	1.12 (1.34, 0.98)
60–64	51.85 (39.46, 65.23)	146.73 (111.67, 184.6)	32.97 (22.75, 46.39)	45.16 (31.16, 63.55)	−3.73 (−4.03, −3.38)	130.02 (100.99, 164.64)	80.96 (62.88, 102.51)	319.25 (258.96, 386.82)	99.75 (80.91, 120.86)	0.68 (0.82, 0.53)
65–69	38.73 (30.5, 47.62)	141.97 (111.78, 174.54)	31.51 (22.63, 42.81)	41.07 (29.5, 55.81)	−3.92 (−4.21, −3.61)	86.11 (67.44, 107.71)	69.67 (54.56, 87.14)	186.87 (152.4, 225.92)	67.75 (55.25, 81.9)	−0.09 (0.04, −0.2)
70–74	24.88 (19.4, 31.52)	132.22 (103.07, 167.52)	22.2 (16.24, 29.47)	41.65 (30.47, 55.29)	−3.66 (−3.85, −3.51)	50.76 (39.58, 64.99)	59.95 (46.75, 76.77)	104.57 (83.69, 130.97)	50.8 (40.66, 63.63)	−0.53 (−0.45, −0.6)
75–79	14.82 (11.57, 18.36)	130.18 (101.7, 161.28)	13.49 (10.16, 17.26)	40.72 (30.69, 52.11)	−3.68 (−3.79, −3.58)	33.04 (26.12, 41.6)	53.68 (42.43, 67.59)	57.47 (46.85, 70.07)	43.58 (35.52, 53.13)	−0.67 (−0.57, −0.77)
80–84	5.57 (4.17, 7.13)	105.19 (78.69, 134.56)	5.74 (4.11, 7.61)	29 (20.78, 38.43)	−4.07 (−4.2, −3.96)	16.65 (12.95, 21)	47.06 (36.61, 59.37)	36.87 (29.97, 45.31)	42.1 (34.22, 51.74)	−0.36 (−0.22, −0.44)
85–89	1.69 (1.3, 2.07)	100.1 (77.21, 122.63)	2.66 (1.98, 3.49)	27.91 (20.76, 36.61)	−4.04 (−4.15, −3.82)	6.69 (5.32, 8.17)	44.24 (35.23, 54.06)	22.09 (18.36, 26.24)	48.31 (40.15, 57.39)	0.28 (0.42, 0.19)
90–94	0.29 (0.23, 0.35)	96.09 (76.04, 115.69)	0.72 (0.54, 0.94)	24.44 (18.33, 32.23)	−4.32 (−4.49, −4.04)	2.05 (1.66, 2.48)	47.74 (38.83, 57.93)	11.65 (9.68, 13.73)	65.11 (54.11, 76.77)	1.01 (1.08, 0.91)
95+	0.02 (0.02, 0.03)	56.75 (41.33, 76.03)	0.12 (0.09, 0.16)	18.83 (13.32, 25.68)	−3.5 (−3.59, −3.44)	0.54 (0.43, 0.65)	53.19 (42.1, 64.14)	4.15 (3.37, 4.9)	76.22 (61.92, 89.93)	1.17 (1.25, 1.1)

ASDR, age-standardized DALYs rate; DALYs, disability-Adjusted Life Years; EAPC, estimated annual percentage change; No., number; UI, uncertainty interval. *: Crude incidence rate in each age group.

### 3.2 Burden and time trends in OUD by China vs. global and OUD by gender in China

**Figure 1:**
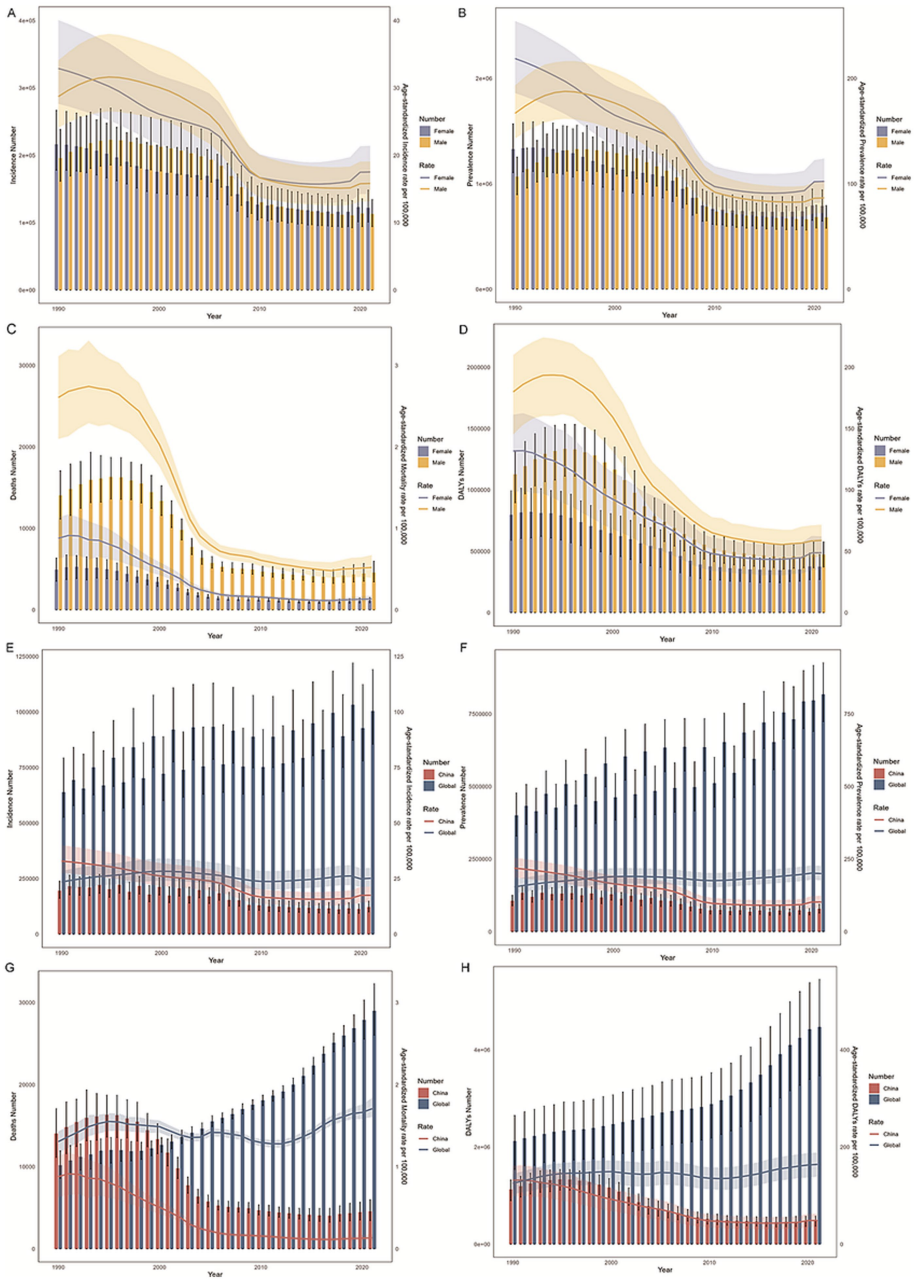
Trends in the burden of opioid use disorder (OUD) in China and globally. **(A,E)** Show the age-standardized incidence rate (ASIR); **(B,F)** Depict the age-standardized prevalence rate (ASPR); **(C,G)** Present the age-standardized mortality rate (ASMR); and **(D,H)** Display the age-standardized disability-adjusted life years rate (ASDR). Gender-specific data for China are shown in **(A–D)**, while global and national comparisons are provided in **(E–H)**.

Between 1990 and 2021, the incidence and prevalence of OUD in China, including both crude and age-standardized rates, were comparable to those in males. By 2021, the disease burden among females had surpassed that of males (Tables 2–5; Figures 1A,B). In contrast, in terms of mortality rate and DALYs, both in absolute numbers and age-standardized rates, the disease burden was higher in females than in males (Figures 1C,D). Overall, the disease burden of OUD in China showed a significant decline, whereas the global burden exhibited varying degrees of increase (Tables 2–5; Figures 1E,H).

### 3.3 Burden and temporal trends of DUDs in the China by age and sex

**Figure 2:**
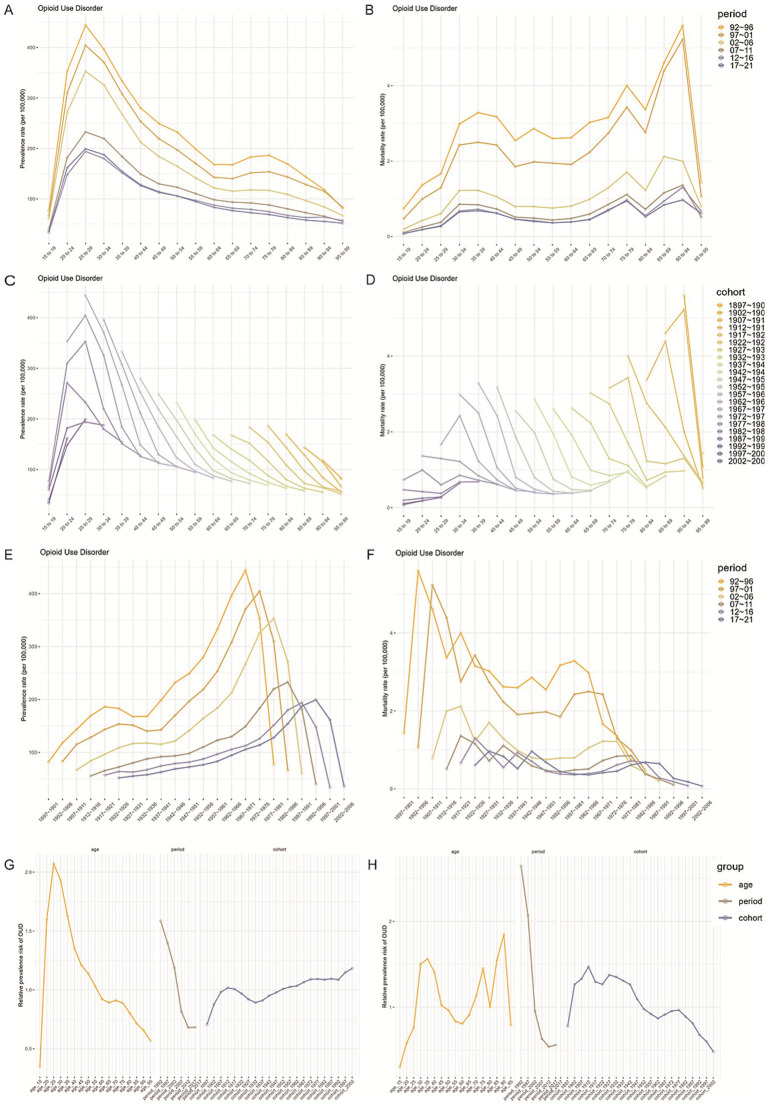
Age, period, and cohort effects on the incidence and prevalence of OUD in China. **(A,B)** Show age-specific incidence and prevalence rates of OUD. **(C,D)** Depict period-based trends, while **(E,F)** display cohort-based trends. **(G,H)** Present estimated age, period, and cohort effects on OUD prevalence and incidence between 1990 and 2021.

The age-period-cohort effects on the prevalence and mortality of OUD are shown in Figure 2 and Table 6, which indicates that the highest prevalence consistently occurred in the 20–24 age group across all periods, with a gradual decline as age increased. This pattern remained stable over time. Significant differences in prevalence were observed between different time periods. For instance, during 1992–1996, there was a noticeable gap between the highest and lowest prevalence rates across age groups. However, in later periods, such as 2017–2021, the overall prevalence was lower (Figure 2A).

**Table 6:** OUD prevalence and mortality relative risks due to age, period, and cohort effects.

Factors	Prevalence			Mortality		
	RR	95%UI	*P*-value	RR	95%UI	*P*-value
Age (years)						
15–19	0.35	0.35–0.35	*	0.30	0.27–0.34	*
20–24	1.60	1.58–1.62	*	0.58	0.53–0.64	*
25–29	2.07	2.05–2.09	*	0.76	0.70–0.82	*
30–34	1.93	1.91–1.95	*	1.50	1.41–1.60	*
35–39	1.63	1.61–1.64	*	1.56	1.48–1.65	*
40–44	1.35	1.35–1.36	*	1.41	1.34–1.47	*
45–49	1.21	1.20–1.22	*	1.02	0.98–1.06	0.32
50–54	1.14	1.13–1.14	*	0.97	0.93–1.00	0.07
55–59	1.03	1.03–1.04	*	0.84	0.80–0.87	*
60–64	0.92	0.92–0.93	*	0.81	0.77–0.85	*
65–69	0.89	0.89–0.90	*	0.91	0.86–0.96	*
70–74	0.91	0.90–0.92	*	1.13	1.05–1.21	*
75–79	0.89	0.88–0.90	*	1.45	1.34–1.57	*
80–84	0.80	0.79–0.81	*	1.00	0.91–1.10	0.97
85–89	0.72	0.70–0.73	*	1.55	1.38–1.74	*
90–94	0.66	0.64–0.67	*	1.85	1.59–2.15	*
95+	0.57	0.54–0.60	*	0.79	0.51–1.23	0.30
Period						
1992–1996	1.59	1.58–1.59	*	2.65	2.55–2.74	*
1997–2001	1.40	1.41–1.43	*	2.07	2.02–2.12	*
2002–2006	1.19	1.19–1.19	*	0.96	0.94–0.97	*
2007–2011	0.82	0.82–0.82	*	0.63	0.62–0.64	*
2012–2016	0.68	0.68–0.68	*	0.54	0.52–0.55	*
2017–2001	0.68	0.68–0.69	*	0.56	0.54–0.58	*
Cohort						
1897–1901	0.71	0.60–0.83	*	0.78	0.22–2.76	0.70
1902–1906	0.88	0.83–0.93	*	1.27	0.93–1.73	0.14
1907–1911	0.98	0.95–1.01	0.25	1.33	1.08–1.65	*
1912–1916	1.02	0.99–1.04	0.19	1.47	1.22–1.77	*
1917–1921	1.01	0.98–1.03	0.57	1.30	1.10–1.53	*
1922–1926	0.97	0.95–0.99	*	1.27	1.09–1.47	*
1927–1931	0.92	0.90–0.94	*	1.38	1.20–1.57	*
1932–1936	0.89	0.88–0.91	*	1.35	1.20–1.53	*
1937–1941	0.91	0.90–0.92	*	1.31	1.17–1.46	*
1942–1946	0.95	0.94–0.96	*	1.26	1.15–1.39	*
1947–1951	0.98	0.97–0.99	*	1.10	1.01–1.19	*
1952–1956	1.01	1.00–1.02	0.07	0.98	0.91–1.05	0.51
1957–1961	1.03	1.02–1.03	*	0.92	0.87–0.97	*
1962–1966	1.03	1.03–1.04	*	0.87	0.83–0.91	*
1967–1971	1.07	1.06–1.07	*	0.91	0.89–0.94	*
1972–1976	1.09	1.09–1.09	*	0.96	0.93–0.98	*
1977–1981	1.09	1.09–1.09	*	0.97	0.94–0.99	*
1982–1986	1.09	1.08–1.09	*	0.89	0.86–0.92	*
1987–1991	1.09	1.09–1.10	*	0.81	0.78–0.86	*
1992–1996	1.09	1.08–1.09	*	0.68	0.63–0.73	*
1997–2001	1.15	1.14–1.16	*	0.60	0.54–0.67	*
2002–2006	1.18	1.17–1.20	*	0.48	0.40–0.59	*

RR, relative risk [RR = exp.(coefficient)]; UI, uncertainty interval. *: *p* value<0.05.

In contrast, mortality rates followed a different pattern, with small peaks in the 35–39, 75–79, and 90–94 age groups. This trend remained consistent across all periods, although there was a noticeable difference during the 1992–2001 period compared to the later years (Figure 2B).

Changes in OUD prevalence and mortality across different time periods are further illustrated in Figures 2C,D. Among younger individuals (20–24 years), early birth cohorts (e.g., 1897–1901) had higher prevalence and mortality rates, while later cohorts (e.g., 1997–2001) had lower rates. However, as age increased, some later cohorts (e.g., those aged 60 and above) showed rising prevalence and mortality. Cohort analysis in Figures 2E,F shows that more recent cohorts (e.g., 2002–2006) experienced a steady increase in both prevalence and mortality across all age groups, while earlier cohorts had lower rates.

### 3.4 Joinpoint regression analysis results

**Figure 3:**
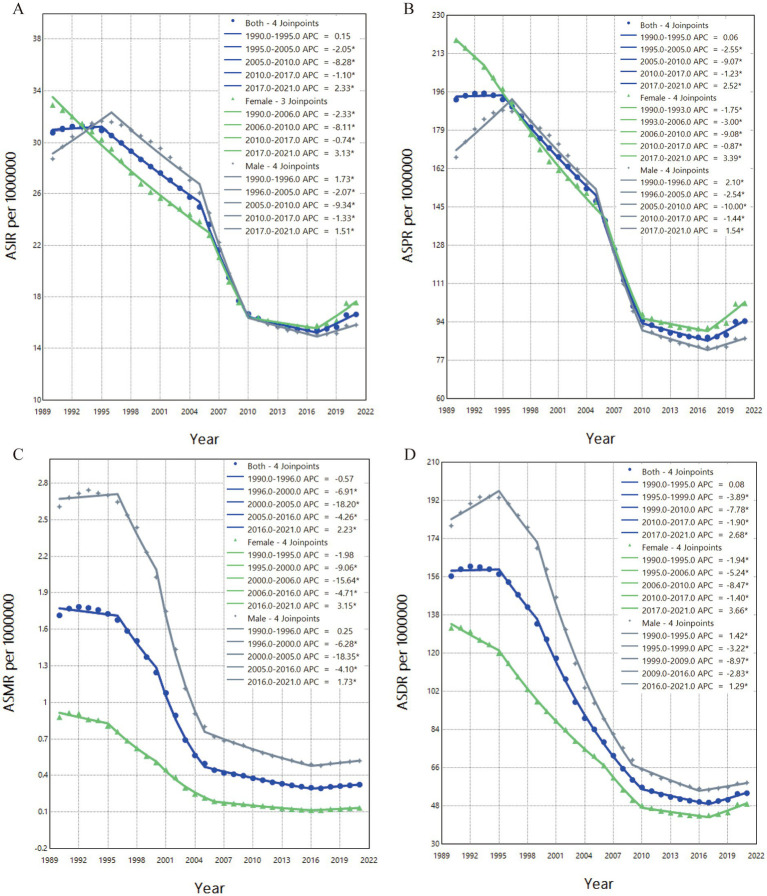
Trends in age-standardized burden indicators and their annual percentage change (APC) for OUD in China, 1990–2021. **(A)** ASIR; **(B)** ASPR; **(C)** ASMR; **(D)** ASDR. APC indicates the annual percentage change for each age-standardized rate.

Based on the Joinpoint regression analysis conducted from 1990 to 2021, the trends in ASRs of OUD in China were further examined by gender. The annual percent change (APC) for each period highlighted significant differences in the OUD trends between males, females, and the overall population. The results showed that, over the observation period, ASIR and ASPR for males first increased, followed by a sharp decline, and finally a slight rise (Figures 3A,B). In contrast, the ASIR and ASPR for females initially showed a continuous decline, reaching their lowest point in 2017, after which a modest increase was observed. The APC for males was 1.51% for ASIR and 1.54% for ASPR, both significantly lower than the APC for females, which were 3.13 and 3.19%, respectively.

The overall trends in ASMR and ASDR for both males and females showed a decline (Figures 3C,D). ASMR and ASDR reached their lowest points in 2016 and 2017, respectively, with the male APCs at 1.73% for ASMR and 1.29% for ASDR, both lower than the female APCs of 3.15 and 3.66%. Subsequently, both ASMR and ASDR experienced a slight increase, with female ASDR showing the largest rise, marked by an APC of 3.66%.

### 3.5 Factors influencing the DALYs in OUD in the China

**Figure 4:**
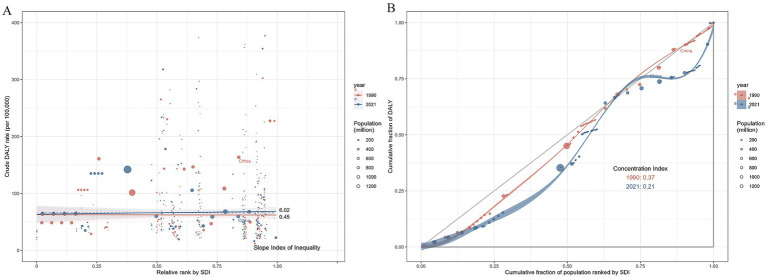
Health inequality trends in OUD-related DALYs across countries, 1990–2019. **(A)** Shows health inequality regression curves; **(B)** Presents concentration curves. Each dot represents a country or region, with larger dots indicating a larger population. DALYs values are plotted against the Socio-Demographic Index (SDI).

Figure 4A shows that the slope index of the DALYs rate between the highest and lowest SDI countries was 0.45 (95% UI: −18.66336, 19.56250) in 1990 and 6.02 (95% UI: −17.71235, 29.74497) in 2021. This change indicates a stronger positive correlation between DALYs and SDI during the observation period, with a notable widening of the DALYs gap between high-SDI and low-SDI countries. The concentration curves and concentration index of DALYs are presented in Figure 4B. In both 1990 and 2021, the concentration curves fell below the equality line, with corresponding concentration indices of 0.37 and 0.21, respectively.

### 3.6 Prediction of OUDs-related burden in China

**Figure 5:**
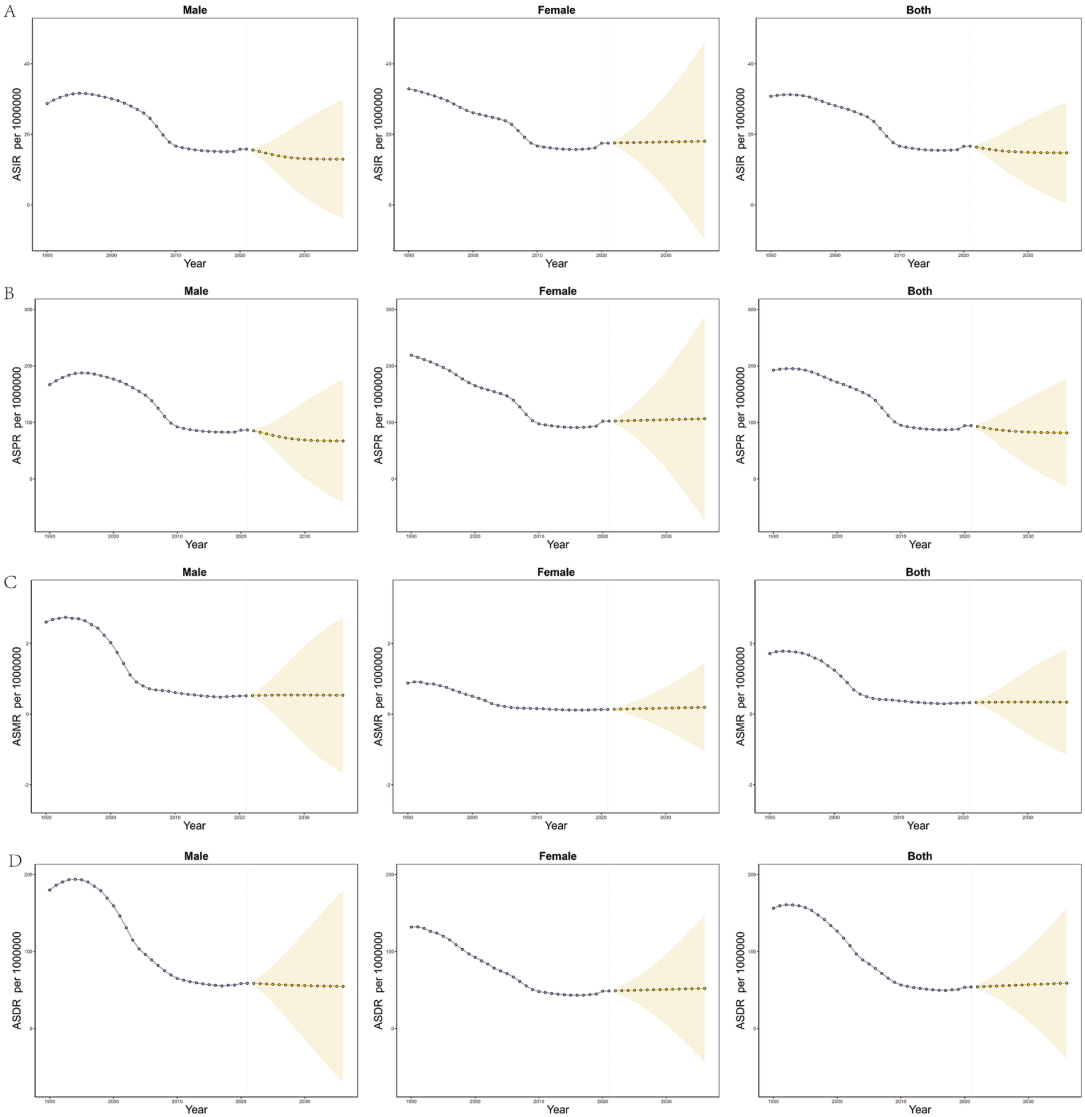
Forecasted trends in age-standardized rates of OUD in China, 2020–2035. **(A)** ASIR; **(B)** ASPR; **(C)** ASMR; **(D)** ASDR. Purple dotted lines represent observed trends during 1990–2019. Yellow dotted lines and shaded regions represent model-based predictions with 95% UI.

Figure 5 presents the projected trends in the burden of OUD in China from 2022 to 2035. The analysis indicates a slight increase in both ASMR and ASDR after 2021, with estimates reaching approximately 0.34 and 59.06 per 100,000, respectively, by 2035. In contrast, both ASIR and ASPR are expected to decline, with projections of about 14.74 and 81.50 per 100,000 by 2035. Notably, the disease burden in females is projected to increase across all four indicators, especially in ASPR, which is expected to rise by 4.27 per 100,000 by 2035 compared to 2021. The disease burden in males is projected to follow the overall trend, with no significant gender differences.

**Figure 6:**
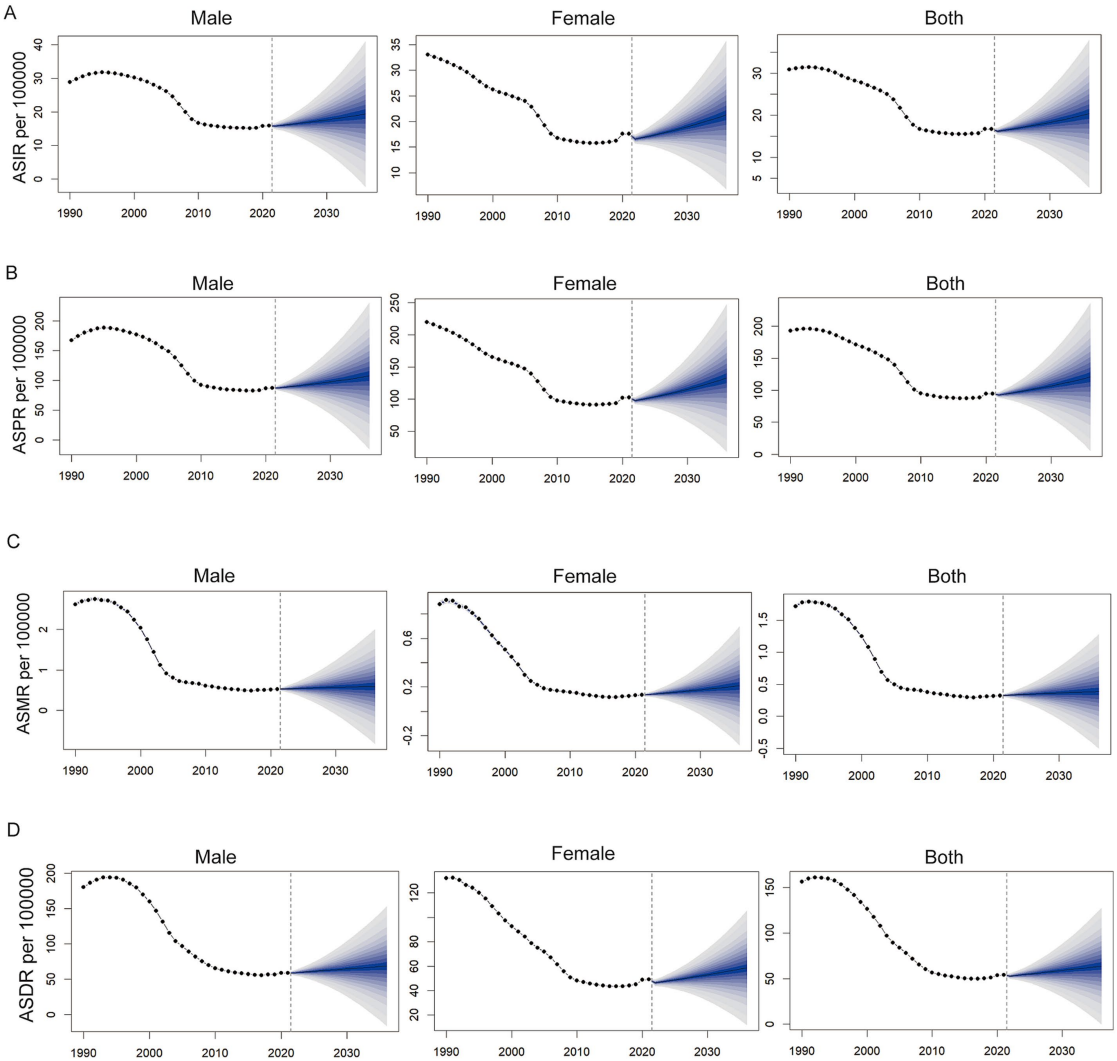
Predicted trends of the burden of opioid use disorder (OUD) in China based on the BAPC model from 2022 to 2035. **(A)** Age-standardized incidence rate (ASIR); **(B)** Age-standardized prevalence rate (ASPR); **(C)** Age-standardized mortality rate (ASMR); **(D)** Age-standardized disability-adjusted life years rate (ASDR) per 100,000. Each panel shows projections for males, females, and both sexes combined.

To further validate the ARIMA model and address its limitations, we conducted a complementary trend analysis using the Estimated Annual Percentage Change (EAPC). The EAPC results were largely consistent with the ARIMA model for females, showing a continued rise in the burden of OUD (Figure 6).

## 4 Discussion

This study, utilizing data from the 2021 GBD study, summarizes the trends in the burden of opioid use disorder (OUD) in China from 1990 to 2021 and projects future trajectories. The findings indicate a global increase in the burden of OUD over the past 30 years. In contrast, during the same period, China experienced a significant reduction in the burden of OUD, particularly after 1995. Notably, the age-standardized mortality rate (ASMR) for OUD in China decreased from 1.71 per 10,000 (95% UI: 1.38, 2.0) in 1990 to 0.32 per 10,000 (95% UI: 0.26, 0.4) in 2021, with an annual percent change (APC) of −5.26 (95% UI: −5.24, −5.06). This decline reflects the substantial progress made in China’s opioid management policies and interventions (16, 17). However, since 2018, there has been a slight rebound in the burden of OUD. Based on our projections, the prevalence and mortality rates of OUD in China are expected to continue rising over the next 25 years from the levels observed in 2021. This trend underscores the urgent need for China’s healthcare system to closely monitor this issue and implement timely, effective strategies to mitigate future challenges.

The analysis based on the age-period-cohort model revealed significant age effects in the incidence of OUD among the 15–24 and 70–74 age groups during the study period, consistent with previous research findings (18). The projections indicate that by 2035, the ASMR for women with OUD is expected to reach 18.06 per million, 7.38 per million higher than that for men. Health inequality analysis found that although the overall health burden gap between regions with different SDI widened with socio-economic development, the regional concentration of disease burden decreased.

For adolescents aged 15–24, this period is crucial for the formation and development of their worldview, and they are more susceptible to environmental factors leading to various psychological issues. Multiple studies have shown that depressive symptoms in adolescents are significant predictors of opioid misuse (19–21), and there may be genetic and neurobiological links between depression and opioid dependence (22, 23). Additionally, the 70–74 age group is a high-risk period for cancer, and the extensive use of analgesics for cancer pain may be a significant reason for opioid addiction in this age group in China (24, 25). Therefore, focusing on and intervening in the mental health and behavior of adolescents, strengthening education and awareness about addictive drugs, and advancing clinical research on opioid-free analgesia are of great significance in reducing the disease burden in China.

The relationship between the SDI and OUD has gradually weakened, and the distribution of the DALYs burden of OUD has become more balanced, indicating that the impact of OUD may have spread to a broader socio-economic group. This diffusion of burden is closely related to the development and improvement of socio-economic and health resources. Socio-economic development has increased the accessibility and availability of opioids in high-SDI regions, further leading to the misuse of prescription drugs (26), while the improvement of health resources has promoted the development of health systems in low-SDI regions, increasing the coverage of medical services but also leading to a rise in disease recognition rates (27), further altering the distribution pattern of the disease burden.

Studies have shown that although the decline in the burden of OUD in women is greater than in men, the overall burden of disease in women in 2018 was still significantly higher than in men, and this trend continued to rise in subsequent years. This gender difference may be related to the role of sex hormones in the pain perception mechanism (28). Literature has proven that testosterone has a protective effect against pain in men (29, 30), while estrogen, due to its fluctuations, weakens the protective effect of stable hormone levels on anti-nociception in women, making them more susceptible to lowered pain thresholds (29, 31). Additionally, the use of psychotropic drugs during pregnancy (32), stronger self-esteem tendencies (4), and susceptibility to mental disorders (33) may all be potential factors contributing to the gender difference in the burden of OUD. Therefore, when formulating policies for women, special attention should be paid to gender differences, focusing on unique risk factors for women in drug use, such as drug use during pregnancy and hormonal fluctuations, providing more personalized medical support and mental health services for women, enhancing health education and self-management capabilities, and encouraging women to actively seek help, which may be effective measures to narrow this gap.

From a clinical perspective, the growing burden of OUD has profound implications for anesthesiology practice (34). Anesthesiologists are required to thoroughly assess the medical history of OUD patients, particularly their opioid use history, prior to surgery (35). When there is a potential for patients to conceal relevant medical information, it becomes essential to evaluate the risk factors and potential complications associated with OUD. This not only increases clinical workload but also places higher demands on the anesthesiologist’s expertise. Furthermore, the pain threshold in OUD patients is altered (36), leading to a reduced response to standard opioid analgesics. As a result, higher starting doses and a wider range of medications are necessary. At the same time, careful attention must be given to potential drug interactions and dose-related complications to ensure anesthesia safety (37). During postoperative recovery, OUD patients require more stringent monitoring and individualized care, underscoring the challenges in healthcare resource allocation as the burden of OUD continues to rise (38). These challenges highlight the critical importance of education and prevention. Strengthening public health advocacy to raise awareness about the risks of opioid misuse, and promoting safe medication practices, are effective strategies to reduce the incidence and spread of OUD. In addition, fostering collaboration between healthcare institutions and communities, as well as actively engaging in the development of public health policies, are essential measures to curb the occurrence and transmission of OUD.

This study has several limitations: First, due to delays in health data reporting and inclusion, and because our analysis is based on GBD data from 3 years ago, recent changes in health status could not be captured. Second, the APC model is based on certain assumptions and may not fully account for all influencing factors (39). However, our approach relies on information theory, particularly bias-corrected AIC statistics, to guide model selection and minimize errors. Third, in some low- and middle-income countries, the lack of epidemiological data and underreporting of cases may impact the accuracy of statistical estimates, potentially leading to an underestimation of the true burden. In addition, the GBD framework may not fully capture the impact of polysubstance toxicity, as deaths involving multiple substances may be attributed to a single cause, introducing some uncertainty. Finally, although we applied ARIMA and EAPC models to forecast trends, their projections for males showed slight differences—likely due to methodological differences: ARIMA captures short-term fluctuations while EAPC reflects average long-term trends. Using both models enhances robustness and provides a more comprehensive picture of the future burden.

## 5 Conclusion

OUD is a critical public health issue in China. The results of our study provide important insights into the burden of OUD in the country, contributing to our understanding of the scale and distribution of this condition. In the future, it is essential to focus on the development of health policies that address the needs of specific populations, including but not limited to individuals of different age groups, genders, and those residing in regions with varying SDI levels. The significant disparities in OUD burden between countries pose challenges to the sustainability and adaptability of China’s public health policies. Given the widespread nature of OUD, it is imperative that policymakers, researchers, clinicians, and the broader society collaborate effectively to implement strategies that will curb the expansion of opioid use disorder in China.

## Data Availability

The original contributions presented in the study are included in the article/supplementary material, further inquiries can be directed to the corresponding author.

## Footnotes

^1^ http://ghdx.healthdata.org/gbd-results-tool

^2^ https://ghdx.healthdata.org/record/ihme-data/global-population-forecasts-2017-2100

## Glossary

AAPC: Average Annual Percent Change
AIC: Akaike Information Criterion
APC: Age-Period-Cohort
ASDR: Age-Standardized Disability-Adjusted Life Year Rate
ASIR: Age-Standardized Incidence Rate
ASMR: Age-Standardized Mortality Rate
ASPR: Age-Standardized Prevalence Rate
ASRs: Age-Standardized Rates
CI: Concentration Index
CRs: Crude Rates
DALYs: Disability-Adjusted Life Years
EAPC: Estimated Annual Percentage Change
GBD: Global Burden of Disease
GDP: Gross Domestic Product
IHME: Institute for Health Metrics and Evaluation
OUD: Opioid Use Disorder
SDI: Social Development Index
SII: Slope Index of Inequality
SUDs: Substance Use Disorders
UI: Uncertainty Interval
YLD: Years Lived with Disability
YLL: Years of Life Lost
